# Supplementary material for: Aggressive conflict used as landmark information in homing desert ants
Source: J Exp Biol. 2026 Sep 7;229(17):jeb252859. doi: 10.1242/jeb.252859 (PMC13615672; doi:10.1242/jeb.252859)
Supplement: Supplementary file 1 [file jexbio-229-252859-s1.pdf]

Dataset 1. Provides all raw data used to create Fig. 1 and 2 in the main text.

Available for download at
https://journals.biologists.com/jeb/article-lookup/doi/10.1242/jeb.252859#supplementary-data
